# In Vitro Digestion of Human Milk: Influence of the Lactation Stage on the Micellar Carotenoids Content

**DOI:** 10.3390/antiox8080291

**Published:** 2019-08-07

**Authors:** Ana A. O. Xavier, Juan E. Garrido-López, Josefa Aguayo-Maldonado, Juan Garrido-Fernández, Javier Fontecha, Antonio Pérez-Gálvez

**Affiliations:** 1 Food Phytochemistry Department, Instituto de la Grasa (CSIC), Campus Universitario, Building 46, 41013 Sevilla, Spain; 2 Unidad de Neonatología, Hospital Virgen del Rocío, 41013 Sevilla, Spain; 3 Institute of Food Science Research (CSIC-UAM), 28049 Madrid, Spain

**Keywords:** breastfeeding, newborn, human colostrum, carotenes and xanthophylls, in vitro digestibility, micellar lipids

## Abstract

Human milk is a complex fluid with nutritive and non-nutritive functions specifically structured to cover the needs of the newborn. The present study started with the study of carotenoid composition during progress of lactation (colostrum, collected at 3–5 d postpartum; mature milk, collected at 30 d postpartum) with samples donated from full-term lactating mothers (women with no chronic diseases, nonsmokers on a regular diet without supplements, *n* = 30). Subsequently, we applied an in vitro protocol to determine the micellarization efficiency of the carotenoids, which were separated by HPLC and quantified by the external standard method. That in vitro protocol is tailored for the biochemistry of the digestive tract of a newborn. To the best of our knowledge, the present study is the first report of carotenoids micellar contents, obtained in vitro. This study reveals, from the in vitro perspective, that colostrum and mature milk produce significant micellar contents of carotenoids despite lipids in milk are within highly complex structures. Indeed, the lactation period develops some influence on the micellarization efficiency, influence that might be attributed to the dynamics of the milk fat globule membrane (MFGM) during the progress of lactation.

## 1. Introduction

Carotenoids are one of the five families of natural pigments widely distributed in the vegetal kingdom, in photosynthetic microorganisms, and some fungi. These pigments are isoprenoid compounds, which contribute to the light-harvesting process and filter harmful light radiations, and display antioxidant activity. According to their structural features, carotenoids are classified as carotenes (pure hydrocarbons) or xanthophylls (oxygenated carotenes), that is, the classical C40 carotenoids, while apo-carotenoids which arise from carotenoid metabolism that shortens the C40 structure, and C30 carotenoids that been described in bacteria complete the main subfamilies of carotenoids. For a more detailed lecture about structural features and biosynthesis of carotenoids, some general overviews are suggested [1,2]. It is the lipophilic nature of these compounds the main feature that marks the mode of interaction with other biomolecules, and the environment where these processes take place. Hence, carotenoids are biosynthesized in fruit and vegetal tissues in the corresponding plastid organelles, which present their own membrane, and behave in a lipophilic environment with other biomolecules. Therefore, the tissues where carotenoids may incorporate and develop further activities should resemble in a similar way either a membrane macrostructure or a lipophilic surrounding.

Additionally, the carotenoids significantly contribute to the nutritional value of natural sources where they distribute, e.g., fruit and vegetables, algae, eggs, and fish [2]. Animals exclusively rely on diet to incorporate these compounds to both internal tissues and systemic circulation where they perform significant functions and biological activities. Hence, almost 10% of the described carotenoids in nature present the structural requisites to transform into vitamin A [3], while all carotenoids exert important functions in immunity, participate in the antioxidant defense system, and are related to a reduced risk of developing chronic diseases [4,5]. These biological activities are the fundamentals to provide evidence of the inverse association between the ingestion of carotenoid-containing fruits and vegetables, or serum carotenoid levels, with risk for various chronic diseases [6,7,8,9,10,11,12].

Taking both topics into account, the study of bioavailability of carotenoids (from digestion to bioactivity) is the key piece of knowledge to establish the actual contribution of carotenoids to human health, from the provitamin A value, with specific significance in some developing countries where the dietary supply of vitamin A sources if often limited [13] to the other biological activities. The considerable research literature regarding the measurement of carotenoids appearing in serum after the ingestion of different carotenoid-rich sources (fruit and vegetables, juices, oils…) either in human beings or animals, was the starting point to learn that efficiency of the bioaccessibility of carotenoids is controlled by several factors that may limit or enhance their final disposition in systemic circulation and inner tissues. Thus, the disseminated work of Castenmiller and West [14] systematically reviewed the factors that influence carotenoid bioaccessibility and bioactivity in vivo, which were condensed in the mnemotechnic term *SLAMENGHI* where each letter represents one variable. It is necessary to remark again that the influence of these factors was initially ascertained from in vivo experiments, while our more recent advancements have made use of in vitro techniques to determine the considerable number of factors that affect bioaccessibility with a similar confidence to the in vivo approach.

**Figure 1:**
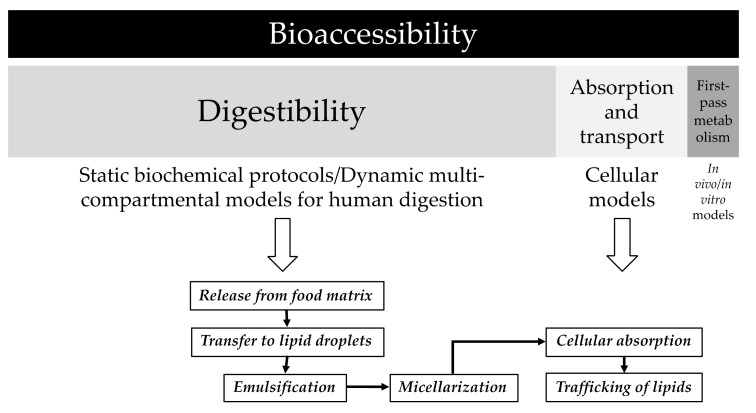
The different contribution of the three sequential steps that yield the bioaccessibility value of carotenoids, including the in vitro models required to measure their efficiency, showing the main molecular events associated to each stage.

Milk is a multifaceted fluid with nutritive and non-nutritive functions and actions, comprising a wide range of molecules in solution, colloidal aggregates, and complex structures such as the milk fat globules. Even cells and microorganisms are observed in this somehow ‘alive’ secretion that impacts the physiology of the newborn at different tissues and at different levels (digestive tract, microbiota, immune system, vision, and cognitive development) [15]. Indeed, breast milk lactation provides both the mother and the infant with significant benefits already documented [16,17,18]. Regarding the quantitative composition, the lipophilic content shows the highest variability so far. Hence, the lipid nutrients represent ca. 45–55% of the total caloric intake, while among lipophilic micro-nutrients counted in human milk, vitamin A is in the higher concentration range (200–600 mg/L) that in addition to carotenoids overcome the concentration of other liposoluble vitamins [19,20]. This is an issue of interest when we realize that the lipophilic vitamins in the fetus are considerably low as the accumulation in the fetus through the placenta during the accretion period is not effective, and the fetal liver stores of lipophilic vitamins are limited [21]. This means that most of the newborn present a deficit of vitamin A after delivery, a deficit that is amended with the lactation. Accordingly, most of the infants are subjected to certain oxidative stress because of the transition from a situation of hypoxia in the uterus (partial O_2_ pressure at 25–35 mm Hg) to the extrauterine environment with partial O_2_ pressure at 100 mm Hg. In addition, the onset of the mitochondrial respiration initiates the concomitant production of reactive oxygen species [22], whereas the immaturity of the endogenous antioxidant system means an additional tendency to increased oxidative stress after delivery [23]. Consequently, there should be a significant interest in determining the bioavailability of those human milk compounds that contribute to relief the oxidative stress of the infant [24]. In vitro digestion protocols have been developed to measure the extent of digestibility, assimilation, and first-pass metabolism of food components, a very active research subject in recent decades [25]. Thus, the digestion protocols have been applied to measure both the bioaccessibility of carotenoids from natural food sources (Figure 1) and the impact of factors in the efficiency of that process [26,27,28,29,30,31]. Indeed, in vitro digestion protocols have been specifically developed for mimicking the gastric conditions of the newborn [32] to determine the bioaccessibility of lipolysis and proteolysis from human, cow, and goat milk; infant formula; and a functional beverage based in whey protein concentrate [33,34,35,36,37].

It is hypothesized that the application of an in vitro protocol that tailors the experimental conditions to those of the gastrointestinal maturity rate of the newborn might provide with significant data regarding the efficiency of the digestion of carotenoids. Hence, the aim of this study was to measure the micellar contents of carotenoids in human milk at two stages of the lactation period: colostrum and mature milk. The application of an in vitro digestion protocol that follows the harmonized international agreement is the core to allow future comparisons among different pools of samples (pre-term, full-term human milk, infant formula). Therefore, the advance in knowledge regarding the accumulation of lipids with significant contributions to the health of the neonate will expand from the state-of-the-art in vitro techniques to the in vivo arena.

## 2. Materials and Methods

### 2.1. Subjects

The study population comprised 30 healthy women, which were recruited within a 12-month period between 2018 and 2019, from a 1200–1500 pregnant population that delivers at the hospital area in Sevilla (Spain). They gave birth to healthy full-term neonates (37–40 weeks). Eligible participants in this study were non-smoking mothers with no chronic disease. In addition, mothers following any special diets, vegetarians, or those taking supplements were not included. Exclusion criteria applied were pathologies and/or infections during the gestation, developmental anomalies in the fetus, or death of the child. Considering our previous studies, 30 volunteers per group would provide enough statistical power to detect (if any) significant differences between colostrum and mature milk of the micellar carotenoid profile [37].

### 2.2. Reagents

Pepsin from porcine gastric mucosa, porcine bile extract, and pancreatin and lipase from porcine pancreas were obtained from Sigma (St. Louis, MO, USA). Solvents (HPLC-grade) were provided by Romyl (Teknokroma, Barcelona, Spain), and the purified water was obtained from a Milli-Q water purification system (Millipore, Milford, MA, USA).

### 2.3. Measures

#### 2.3.1. Milk Collection

The samples were collected at the Unidad de Neonatología of the Hospital Universitario Virgen del Rocío (Sevilla, Spain). Full-term mothers donated colostrum at 3–5 days postpartum or mature milk at 30 days postpartum. Milk samples were obtained by collection of the total milk volume of one breast during one milk expression session into a polypropylene bottle. The samples were transported directly to the laboratory and stored at 4 °C. Subsequent analyses of the samples (carotenoid extraction and in vitro digestion) were performed within 1–2 days after collection.

#### 2.3.2. In Vitro Digestion of Human Milk

The experimental conditions described by Ménard et al. [32] were applied with slight modifications. The in vitro protocol does not include the oral phase, while the gastric and intestinal steps mimics the digestive conditions of full-term infants. Colostrum or mature milk sample (6 mL) was mixed with 3.5 mL of gastric fluid and incubated for 60 min under magnetic stirring in a water bath at 37 °C. The composition of the gastric fluid was 94 mM NaCl and 13 mM KCl at pH 5.3 (HCl 1 M). Enzymes of the gastric fluid were pepsin (268 U/mL) and lipase (19 U/mL). At the end of the gastric phase, sample was cooled in water, pH adjusted to 6.6 and mixed with 5.5 mL of intestinal fluid, and the resulting cocktail incubated at 37 °C with magnetic stirring for 60 min. The intestinal fluid was composed of 164 mM NaCl, 10 mM KCl, 85 mM NaHCO_3_, and bile extract at 3.1 mM bile salt concentration at pH 7. Porcine pancreatin, with 90 U/mL of lipase activity, was added to the intestinal fluid. The upper micellar fraction [38,39] was isolated from digested sample by centrifugation (12,000× *g*, 5 min, 4 °C) in an Avanti^TM^ J-25 centrifuge (Beckman Coulter^TM^, Brea, CA, USA) equipped with a Beckman model JA-25.50 rotor (Kildare, Ireland). The micellar fraction was collected and used for measurement of the micellar carotenoid content. Three replicates of the in vitro digestion procedure for each sample (colostrum or mature milk) were carried out.

#### 2.3.3. Extraction of the Carotenoid Fraction

The experimental conditions previously described by Ríos et al. [37] were slightly modified for extraction of carotenoids from human milk. Sample (3 mL) was mixed with 3 mL of KOH:methanol (20% *w*/*v*), and the mixture was incubated for 1 h, at 25 °C. After hydrolysis, 6 mL of methanol were added, and the mixture was vortex-mixed for 2 min and cooled at −20 °C for 20 min. Subsequently, the cooled mixture was centrifuged at 10,000× *g* and 4 °C for 5 min, discarding the upper layer. Diethyl ether (5 mL) and hexane (2 mL) were added to the pellet and vortex-mixed for 2 min. Then, 5 mL of NaCl 10% (*w*/*v*) was added, and the sample was vortex-mixed again for 2 min. After centrifugation (10,000× *g* at 4 °C for 5 min), the organic layer was washed with water until neutral pH was reached. The organic extract was evaporated to dryness in a rotatory evaporator at 25 °C, and the residue was dissolved in 0.25 mL of acetone. Carotenoids from micelles were extracted following the same procedure. The final extracts were filtered through a 0.22 μm filter and stored at −20 °C until analysis by HPLC, which was performed within 1 week. The extraction and HPLC analysis of carotenoids was performed under diminished light and avoiding excessive contact with air.

#### 2.3.4. Quantification of Carotenoids in the Carotenoid Extracts

Carotenoid extracts from human milk samples and from micellar fraction were analyzed using a Jasco HPLC (Easton, PA, USA) equipped with quaternary pump (model PU-2089-plus), autosampler (model AS-2055-plus), and diode array detector (MD-2010-plus). Chromatographic data were acquired and managed using the Jasco ChromPass Chromatography Data System software (version 1.8.6.1). Carotenoids were separated on a Luna (Phenomenex, Torrance, CA, USA) C18 column (250 × 4 mm, 5 μm particle size), which was stabilized at 25 °C, using a linear gradient of acetone/water, from 75:25 (*v*/*v*) to 95:5 in 5 min, hold 95:5 for 7 min and to 100:0 in 3 min, maintaining this proportion for 10 min, and going back to 75:25 in 5 min. Flow rate was set at 1.5 mL/min and 100 μL of sample was injected. The UV–visible absorption spectra were acquired between 200 and 600 nm and the chromatograms processed at 450 nm. The carotenoids were identified according to elution order on C18 column and characteristics of UV–visible spectrum (λmax, spectral fine structure (% III/II), and peak *cis* (if present) intensity (% AB/AII)), as compared to standards and data available in the literature [40]. Stock solutions were prepared for β-carotene, β-cryptoxanthin, lutein, and lycopene at a concentration of approximately 25 mg/L. Once the exact concentration was determined, working stock solutions for external calibration curves were prepared at 5 concentration levels ranging 0.150–10 mg/L. Lutein and zeaxanthin, as well as their *cis* isomers were quantified as a single peak, while α- and β-carotene, and their *cis* isomers, were quantified as a single peak

#### 2.3.5. Determination of the Lipid Content

The lipid content of human milk samples was determined according to the solvent extraction procedure and then by gravimetry [41].

### 2.4. Ethics Approval

Participants provided informed consent for inclusion before they participated in the study, which was conducted in accordance with the Declaration of Helsinki. The study protocol was approved by the Ethics Committee of the Hospital Universitario Virgen del Rocío (AGL2017-87884-R).

### 2.5. Statistical Analysis and Calculation

Efficiency of the micellarization of carotenoids (%) was determined as the ratio of individual carotenoids in micelles to their corresponding content in the milk samples. Due to the non-normality of the distribution of the content of individual carotenoids (Shapiro–Wilk test, *p* < 0.05), the data were analyzed using a non-parametric statistical procedure in the SPSS software (IBM^®^ SPSS^®^ Statistics version 24, IBM, New York, NY, USA). Hence, data are reported as the median, including 25th and 75th percentiles. Considering the sample size and the heteroscedasticity of the variances, we applied the Friedman test to establish whether the carotenoid contents within each group of samples, both in the human milk and in the micellar fraction, were significantly different or not. Then, we applied the Wilcoxon test to analyze which carotenoids contents were significantly different within each group of samples. To analyze whether the micellar contents were significantly different between colostrum and mature milk, we applied the Kruskal–Wallis test. Subsequently, the Mann–Whitney test was applied to analyze the differences among the micellar contents of colostrum and mature milk samples, and to compare the micellar contents within each type of human milk samples. The significance test was set at *p* < 0.05.

## 3. Results

**Figure 2:**
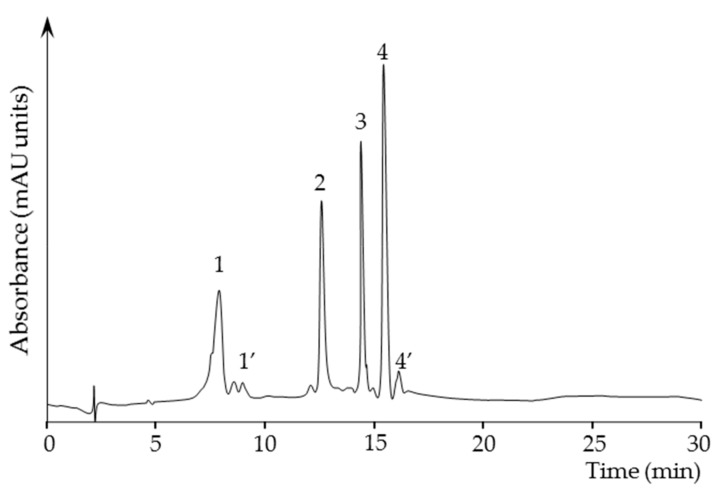
HPLC trace at 450 nm of the carotenoids extracted from the micellar fraction of a human mature milk sample digested in vitro. Carotenoid identification is as follows: 1, zeaxanthin + lutein; 1′, *cis*-isomers of zeaxanthin + lutein; 2, β-cryptoxanthin; 3, lycopene; 4, α+β-carotene; 4′, *cis*-isomers of α+β-carotene.

Figure 2 shows a chromatogram trace of the carotenoids extracted from the micellar fraction obtained after the in vitro digestion of a human mature milk sample. Carotenoids in human milk samples followed the already established trend for the transition from colostrum to mature milk (Table 1). A high content in colostrum for all the carotenoids is significantly reduced in the mature milk, which represents the 13% of the initial colostrum content. This sharp decline is not correlated with a reduction of the fat content as colostrum and mature milk showed total milk fat content in the same range (30–40 mg/mL). Regarding the amounts of the individual carotenoids, lycopene was the carotenoid with the lowest presence in the analyzed samples at both lactation stages, then α+β-carotene (plus *cis* isomers), β-cryptoxanthin and finally the sum of zeaxanthin and lutein (plus *cis* isomers). Xanthophylls (X) and carotenes (C) were equally distributed in colostrum, while mature milk showed a X to C ratio higher than 1.

**Figure 3:**
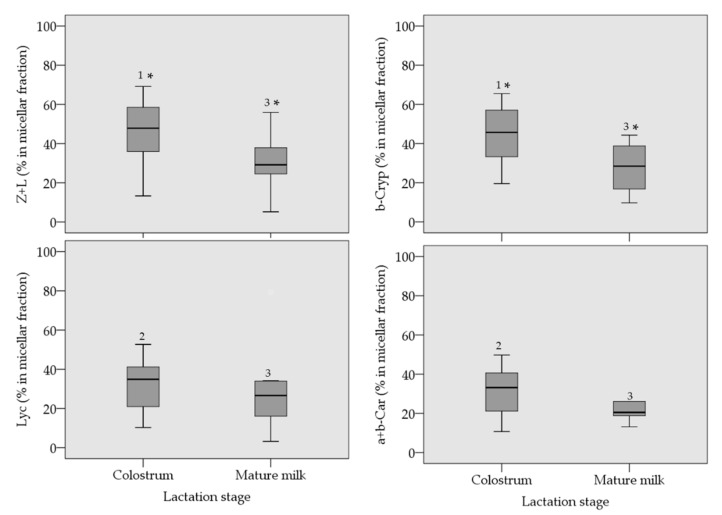
Micellar carotenoid contents (median percentage values ± 75th percentile) in colostrum and mature milk. Data were analyzed using non-parametric statistical procedure. Data denoted with the same number were not significantly different, both for colostrum and mature milk (Wilcoxon test, *p* > 0.05). Data denoted with an asterisk were significantly different (Mann–Whitney test, *p* < 0.05).

Figure 3 depicts the micellar contents of carotenoids in the digested samples of colostrum and mature milk. Regarding the micellar contents in colostrum samples, no significant differences were observed within the X, or within the C. However, when the micellar content of both groups of carotenoids are compared, significant differences were observed, with the X reaching a micellar efficiency of ca. 50%, and below 40% for the C. In the case of the micellar contents of carotenoids after the in vitro digestion of mature milk, no significant differences were observed neither within the X, and within the C as well as in the comparison of the micellar content of X with those of C, reaching both groups of carotenoids a micellar efficiency at the 25–30% range. Finally, the micellar efficiency is significantly higher in colostrum for zeaxanthin + lutein, and β-cryptoxanthin, in comparison with the corresponding micellar contents in mature milk, while lycopene and α+β-carotene are equally micellarized independently of the lactation state.

## 4. Discussion

As new data are added to literature regarding the micellarization efficiency of carotenoids from natural food sources, a sort of database is built that allows to compare the efficiency of that process in different foods, and how it is influenced by factors such as the food structure, food matrix and processing features, as well as by those physiological issues that might be reproduced with the in vitro digestion protocol [25]. Thus, according to the results provided with this study, human colostrum yields micellar carotenoid contents like those provided by whole and semi-skimmed milk and yogurt (45%) enriched with a water-soluble lutein formulation [39], and higher than the micellar carotenoid content observed after the in vitro digestion of a puree of cooked vegetables (29–37%) containing spinach, carrot, and tomato [42]. Indeed, the values observed in this study are close to those reported (58–78%) for the in vitro digestion of cooked durum wheat pasta, and cooked pasta containing eggs [27]. It should be noted that although the in vitro protocols applied in the cited studies present slight modifications among them, the experimental approach is basically the same [42]. However, the data presented in the study of Lipkie et al. [43] for micellarization efficiency of carotenoids from mature human milk and infant formula samples are significantly lower than those presented in this study. Although the authors made slight modifications to reduce the sample volume in digesta and reproduce the biochemical gut conditions, the observed differences demonstrate a need for a consensus protocol, so that interlaboratory comparisons are feasible and reproducible.

Therefore, we applied an in vitro model that tailors the experimental conditions of the digestion to those of an infant [32], although the international consensus already published is generally followed [44]. In that protocol for ‘infants’, the ratio to meal value is higher than in the ‘adult’ protocol, while the enzymatic capability contained in the digestive fluids is considerably lower. Consequently, the comparison of the data presented in this study with those published in the literature is not straightforward. Certainly, it could be assumed that efficiency of carotenoid micellarization from colostrum and mature milk is higher than it should be, as the sequence of physicochemical processes involved in the digestion and absorption of lipids (Figure 1) is highly dependent of the action of digestive secretions.

Another issue that should be discussed when presenting data acquired with in vitro techniques is their correlation with in vivo data, if available, or their predictive power to the real in vivo scenario. In addition, that comparison among in vitro and in vivo results is useful to reinforce the application of the in vitro protocol or to introduce modifications to obtain a better correlation. There are some in vivo data regarding the assimilation of carotenoids from human milk and infant formula. Thus, the serum lutein content of breastmilk-fed infants increased from the baseline level after delivery to reach almost six-fold higher serum lutein values than in formula-fed infants [45]. The same study showed that to obtain similar serum lutein levels as in the breastmilk-fed group, the formula-fed group required a supplementation in the formula with 4-times more lutein than the content observed in the breastmilk. This study denoted the efficiency of the digestive system of the infants to assimilate carotenoids (lipids) from the structured milk fat globules, and that at equal lutein concentration in the fed, the efficiency is higher from human milk. Our data agreed with this efficiency as it was expressed above. Efficiency regarding the micellarization process of colostrum, which for lutein and zeaxanthin is in the highest range when compared with other carotenoid food sources. Application of the in vitro digestion protocol to infant formula would complete the vision of the correlation of in vitro with the in vivo data. Another study showed that plasma carotenoids of infants fed with breastmilk are in the same concentration range, independently of whether they belong to the X or to the C groups [46]. Thus, β-cryptoxanthin, lycopene, and β-carotene in the plasma of breastfed children was ca. 20 μg/L at 2–6 weeks after delivery. Although the authors stated that the data present statistical differences in concentration, our results point to a similar accessibility of carotenoids from mature milk (Figure 3), e.g., they potentially contribute equally to the pool of plasma carotenoids.

Regarding the change of behavior observed in the lactation stage from a higher in vitro micellarization of X in colostrum to the same micellar contents for X and C after digestion of mature milk, this issue could be related with changes in the lipid and protein composition of the MFGM during the lactation period. Lipids from the mammary gland tissue are secreted in milk as milk fat globules enveloped by a three-layered membrane, the MFGM arising from the cellular membrane of the mammary epithelium [47]. That macrostructure, which contains a wide range of polar lipids and membrane associated proteins, envelopes a core rich in triacylglycerides and lipophilic micronutrients. Therefore, the activity of enzymatic secretions may take different rates to access the core of triacylglycerides where most of the carotenoids are accumulated, depending on the composition of the MFGM. The dynamics of the composition of the MFGM has been demonstrated over the course of lactation for the protein profiles [48], while the evolution of the lipids has not been fully characterized so far [49]. Consequently, the influence of the composition of the milk fat globule membrane in the extent of lipolysis is a feasible hypothesis that deserves further attention. One limitation of this study is the lack of in vivo data regarding the bioaccessibility of carotenoids in the newborn to correlate with the in vitro results, which would additionally support the experimental conditions applied for digestion of the colostrum and mature human milk samples.

## 5. Conclusions

To the best of our knowledge, the present study is the first report of carotenoids micellar contents in both colostrum and mature human milk after the application of an in vitro protocol, which is tailored for the biochemistry of the digestive tract of a newborn. Our study reveals, from the in vitro perspective, that colostrum and mature milk produce significant micellar contents of carotenoids despite lipids in milk being within highly complex structures. Indeed, the lactation period develops some influence in the micellarization efficiency, influence that might be attributed to the dynamics of the MFGM during the progress of lactation. These results may serve for comparative aims for future studies regarding digestibility of human milk and the influence of other factors far from the lactation period. This new knowledge is a challenge for food industry that could be achieved with the experimental work to develop in this proposal. The design of infant formula for specific infant sub-populations may apply the experimental protocol outlined here to test the efficiency of the digestibility of the supplemented nutrients included in the formulation. Indeed, our study encourages closely mimicking, as far as possible, the architecture of the complex lipid structures presented in human milk, which are lacked in infant formula, considering the digestive efficiency they present.

## Figures and Tables

**Table 1:** Carotenoid content in colostrum and mature human milk (*n* = 30). Data are expressed in ng/mL.

Carotenoid	25th Percentile	Median ^1^	75th Percentile
		Colostrum ^2^	
Zeaxanthin + lutein ^3^	190	349	664
β-cryptoxanthin	195	285	464
lycopene	114	224	371
α+β-carotene ^4^	133	241	265
		Mature Milk ^5^	
Zeaxanthin + lutein ^3^	26.7	43.9	67.0
β-cryptoxanthin	9.51	37.6	62.7
lycopene	6.79	11.6	19.8
α+β-carotene ^4^	6.45	41.8	51.5

^1^ Data are significantly different when colostrum and mature milk values are compared (Mann–Whitney test, *p* < 0.01). ^2^ Data were not significantly different (Friedman test, *p* > 0.05). ^3,4^ Data include the *cis* isomers when observed. ^5^ Data were not significantly different (Friedman test, *p* > 0.05) except for lycopene.
